# Recent Developments in Clinical Applications of Mesenchymal Stem Cells in the Treatment of Rheumatoid Arthritis and Osteoarthritis

**DOI:** 10.3389/fimmu.2021.631291

**Published:** 2021-03-08

**Authors:** Joel Jihwan Hwang, Yeri Alice Rim, Yoojun Nam, Ji Hyeon Ju

**Affiliations:** ^1^College of Public Health and Social Justice, Saint Louis University, St. Louis, MO, United States; ^2^Catholic Induced Pluripotent Stem Cell Research Center, College of Medicine, The Catholic University of Korea, Seoul, South Korea; ^3^Division of Rheumatology, Department of Internal Medicine, Seoul St. Mary's Hospital, Institute of Medical Science, College of Medicine, The Catholic University of Korea, Seoul, South Korea

**Keywords:** mesenchymal stem cell, rheumatoid arthritis, osteoarthritis, cartilage, cell therapy

## Abstract

Mesenchymal stem cell (MSC) therapies have been used as cell-based treatments for decades, owing to their anti-inflammatory, immunomodulatory, and regenerative properties. With high expectations, many ongoing clinical trials are investigating the safety and efficacy of MSC therapies to treat arthritic diseases. Studies on osteoarthritis (OA) have shown positive clinical outcomes, with improved joint function, pain level, and quality of life. In addition, few clinical MSC trials conducted on rheumatoid arthritis (RA) patients have also displayed some optimistic outlook. The largely positive outcomes in clinical trials without severe side effects establish MSCs as promising tools for arthritis treatment. However, further research is required to investigate its applicability in clinical settings. This review discusses the most recent advances in clinical studies on MSC therapies for OA and RA.

## Introduction

The first study on mesenchymal stem cells (MSCs) was published in 1966 by Fridenshtein et al., who cultured bone-forming cells from guinea-pig bone marrow and spleen cells (1, 2). Subsequent studies have characterized MSCs as clonogenic progenitor cells capable of differentiating into mesoderm-derived cells such as osteoblasts, chondrocytes, and adipocytes (1, 3–5). The term “mesenchymal stem cell” was first used in 1991 to represent cells originating from embryonic mesodermal tissues (5, 6). While, MSCs imply mesenchymal “stem” or “stromal” cells at the same time, it is suggested only to refer progenitor cells with self-renewal and differentiation ability as “mesenchymal stem cells.” Mesenchymal stromal cells, on the other hand, refer to a bulk population of cells with immunomodulatory and homing properties. Some researchers, however, have recently argued that MSCs should be renamed “medicinal signaling cells” because these cells secrete therapeutic regenerative bioactive factors to stimulate the site- and tissue-specific resident stem cells of patients rather than differentiating into tissue-producing cells (7). Nevertheless, the wide clinical potential of MSCs, which ranges from repairing simple tissue tears to regulating immunological diseases, remains to be fully elucidated (2). Hence, researchers worldwide continue to explore the applications of MSCs. Notably, there were 1,043 trials involving 47,548 patients conducted between 2011 and 2018 (2).

Although the criteria for defining human MSCs are not concrete and subject to changes, most researchers agree on the three defining characteristics of human MSCs established by the International Society of Cellular Therapy (ISCT) (8, 9). The first general characteristic of MSCs is plastic adherence where cells with clonal expansion ability can be maintained for several passages in plastic culture dishes while excluding the subpopulation of cells with hematopoietic functions (9, 10). This characteristic is generally believed to encompass all types of MSCs without any exceptions. The second feature of human MSCs is the unique set of positive and negative surface markers expressed on these cells; the ICST has proposed CD105 (endoglin), CD73 (ecto-5′-nucleotidase), and CD90 (Thy-1) as the surface markers of MSCs. In contrast, MSCs lack the expression of hematopoietic and endothelial markers, such as CD45, CD34, CD14, CD11b, CD79α, CD19, and human leukocyte antigen (HLA)-DR (8, 9). Although the list proposed by the ICST is generally agreed upon by researchers, this criterion is the most disputed; some researchers regard CD34, CD45, and CD14 as negative markers, while STRO-1, CD29, CD73, CD90, CD105, CD106, CD166, CD146, and CD44 are considered positive markers (11). Multiple studies have also discovered variations from this criterion. For instance, fractions of adipose tissue derived-MSCs (AT-MSCs) were observed to express CD34 when insulin-like growth factor 1 (IGF-1) was added to the culture media (12). Moreover, the expression of the negative marker HLA-DR was upregulated in murine and human MSCs after exposure to interferon-γ (IFN-γ) (13, 14). Hence, positive and negative surface markers are not widely used to classify *in vitro*-expanded MSCs, and further research is needed to clarify this criterion. The final and most defining characteristic of MSCs is the ability to differentiate into osteoblasts, adipocytes, and chondroblasts *in vitro* (8). As mentioned earlier, the differentiation potential of MSCs into various cell lineages was a factor in their early classification as a type of stem cell and remains one of their key traits (6).

The first clinical studies involving MSCs assessed their therapeutic potential in hematopoiesis, osteogenesis imperfecta, Hurler syndrome, and metachromatic leukodystrophy (15–19). These studies provided initial safety assessments for MSCs and encouraged further research to thoroughly examine their clinical efficacy (15). Recent decades have seen clinical trials conducted on these cells, especially for autoimmune, neurodegenerative, cardiovascular, and bone and cartilage diseases (20). However, the number of approved MSC treatments worldwide remains limited. Asian countries have approved more MSC treatments than other countries; South Korea has approved four MSC therapies, whereas Japan and India have each approved one (Table 1).

**Table 1:** A list of approved cell therapy products around the world and a graphical image of countries where MSC therapies are approved and clinically used.

**Name**	**Country**	**Product description**	**Date of market approval**	**Current status**
Prochymal (MESOBLAST INTERNATIONAL SARL)	Canada	Allogeneic *ex vivo*-cultured adult human mesenchymal stromal cells for the management of acute graft-vs.-host disease (aGVHD) in pediatric patients	May 2, 2015	The product was never marketed in Canada
Stempeucel^®^ (Stempeutics Research)	India	*Ex vivo*-cultured adult allogeneic mesenchymal stromal cells for the treatment of critical limb ischemia due to thromboangiitis obliterans (Buerger disease)	May 2016	On the market, limited release (200 patients on a cost recovery basis), post-market surveillance study required
Temcell HS (JCR Pharmaceuticals Co. Ltd.)	Japan	Allogeneic mesenchymal stromal cells for the treatment of aGVHD	September 2015	On the market
Prochymal (Osiris Therapeutics Incorporated)	New Zealand	Allogeneic *ex vivo*-cultured adult human mesenchymal stromal cells indicated for the rescue of patients with NLT 6 month to 17 year of age with aGVHD, refractory to treatment with systemic corticosteroid therapy or other immunosuppressive agents	June 14, 2012	Approval lapsed
NEURONATA-R^®^ (Corestem, Inc.)	South Korea	Autologous bone marrow mesenchymal stromal cell therapy for amyotrophic lateral sclerosis	July 30, 2014	Orphan product
Cupistem^®^ (Anterogen)	South Korea	Autologous adipose tissue-derived mesenchymal stromal cell therapy for Crohn's fistula	January 18, 2012	Covered by insurance as of 2014, orphan product
CARTISTEM^®^ (Medipost Co., Ltd.)	South Korea	Human umbilical cord blood-derived mesenchymal stromal cells for the treatment of knee articular cartilage defects in patients with osteoarthritis (ICRS grade IV)	January 18, 2012	On the Market
Cellgram^®^-AMI (Pharmicell Co., Ltd.)	South Korea	Autologous bone barrow-derived mesenchymal stromal cells for patients with acute myocardial infarction (left ventricular ejection fraction improvement)	July 1, 2011	Name at time of approval was Hearticellgram^®^-AMI, on the market
Ixmyelocel-T (Vericel)	USA	Autologous expanded multicellular (mesenchymal cells, monocytes, and alternatively activated macrophages) product for patients with advanced heart failure due to ischemic dilated cardiomyopathy	May 10, 2017	Orphan product
Alofisel^®^ (Takeda Pharma A/S)	European Union	Allogenic adipose tissue-derived mesenchymal cells used for complex anal fistulas in adults with Crohn's disease	March 23, 2018	Orphan product
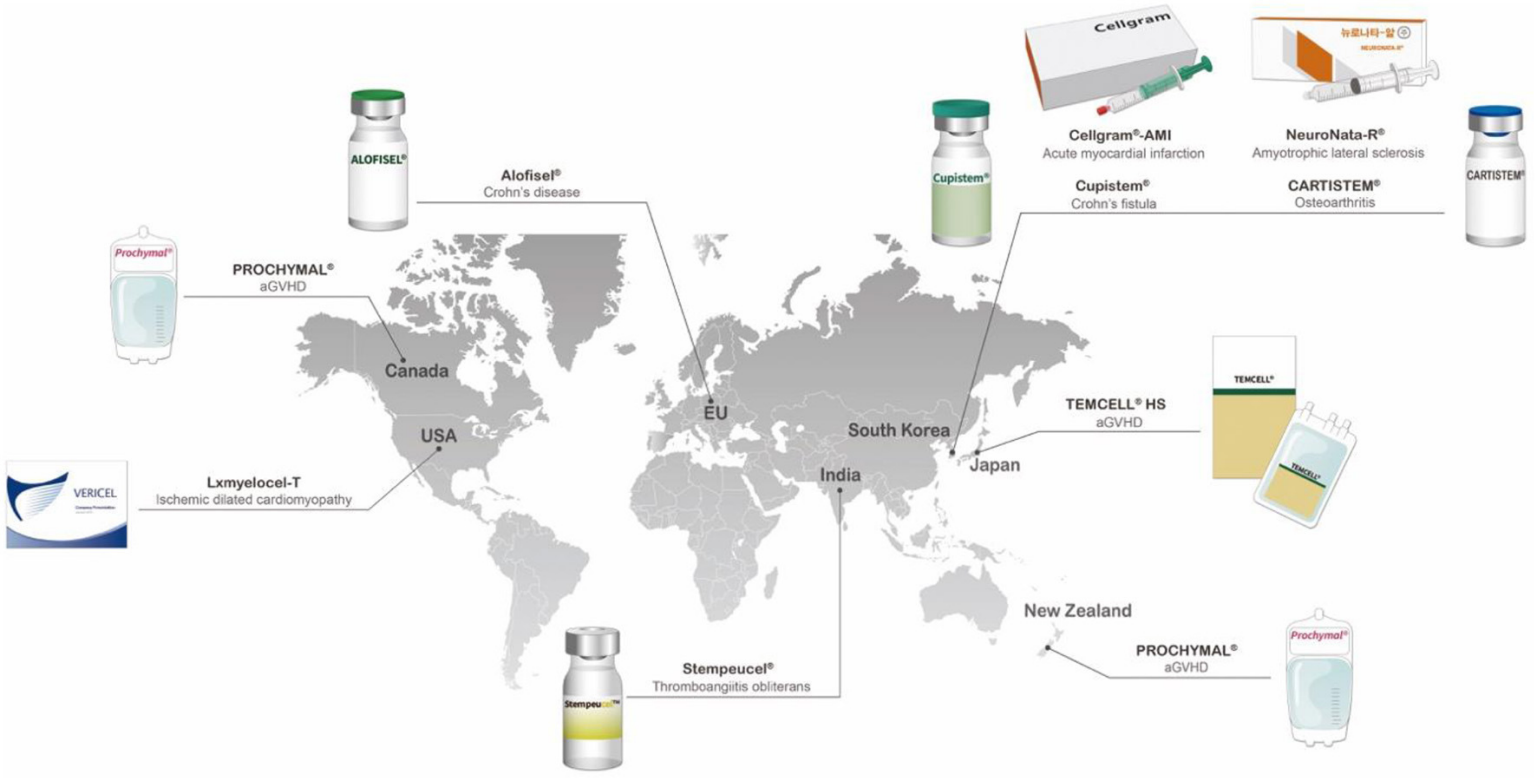				

*Asian countries have approved more MSC treatments than other countries. South Korea has approved four MSC therapies, while Japan and India have each approved one*.

## Human Tissues Containing MSCs and the Various Potentials of These Cells

**Figure 1:**
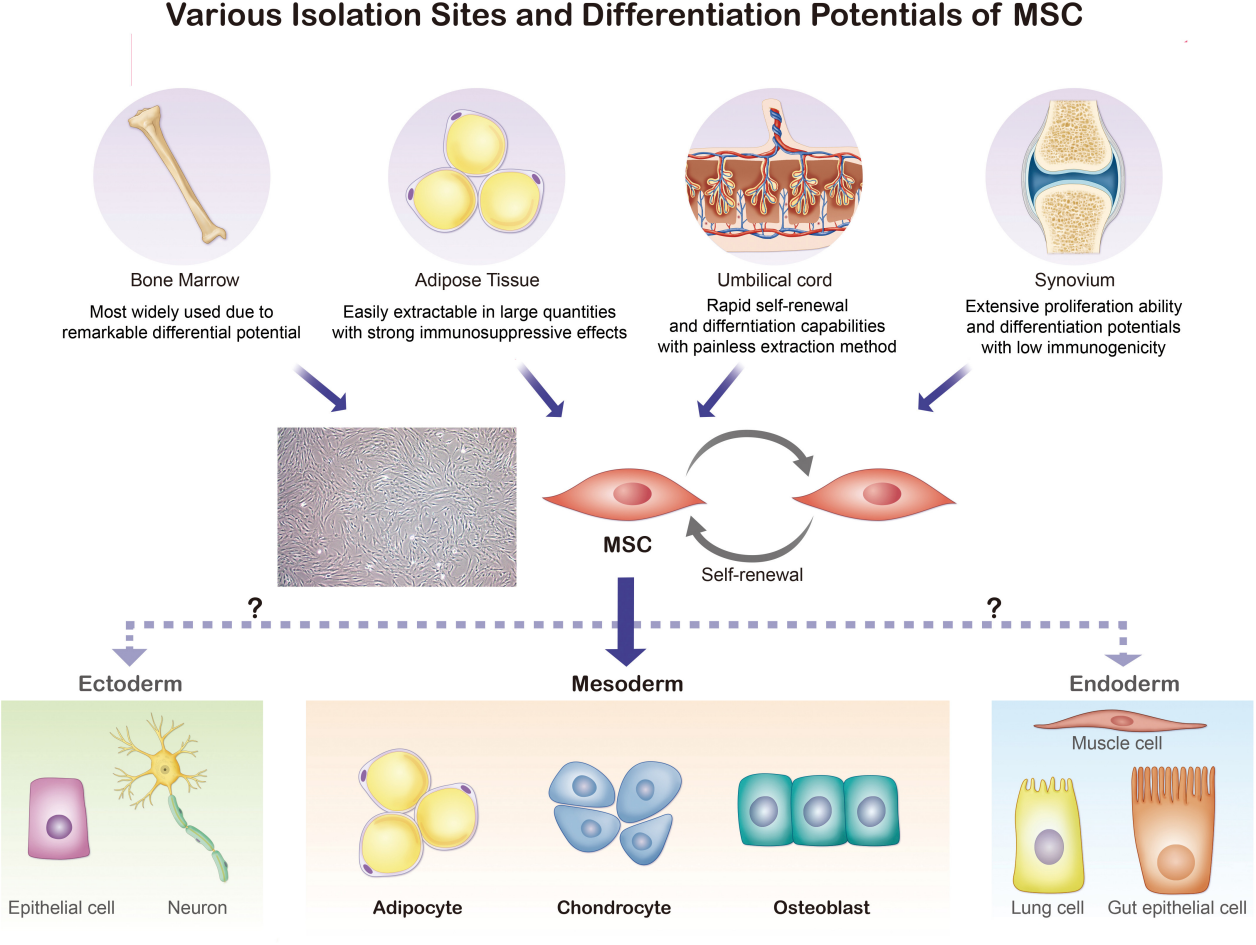
Various isolation sites and differentiation potentials of MSCs. Bone marrow, adipose tissue, umbilical cord, and synovium are common sites for MSC extraction. The isolated MSCs can be differentiated into adipocytes, chondrocytes, myocytes, and osteoblasts.

The ISCT MSC committee recommends not to use the term mesenchymal “stem” cells unless there is rigorous *in vitro* and *in vivo* functional evidence that can provide the self-renewal and differentiation ability (9). While MSCs are found in various parts of the human body, MSCs isolated from the bone marrow (BM), umbilical cord blood, periosteum, dental pulp, adipose tissue, and growth plate were confirmed to have stem cell-like properties [Figure 1; (21–23)]. In this section, we will discuss four sites where MSCs are frequently found and used for treatment of arthritic diseases: the bone marrow, umbilical cord, adipose tissue, and synovial membrane (24). To select a suitable MSC source for treatment, both advantages and disadvantages of MSC acquisition including the potential side effects and limitations (e.g., cell quality, number, and the difficulty and invasiveness of the isolation process) must be considered (25).

Bone marrow was the initial extraction site used by Fridenshtein et al. (1). After years of animal studies, the isolation and expansion of human bone marrow-derived MSCs (BM-MSCs) in culture were first conducted in 1992 (2, 26). With the safety and effectiveness of BM-MSCs confirmed through multiple clinical trials, they have become the most widely-used source of MSCs characterized by remarkable differentiation potential (27). However, there are several limitations of BM-MSCs. Most importantly, the yields, along with the differentiation and repair potential, are heavily dependent on the donor characteristics, such as disease condition and age (25). Furthermore, BM-MSC harvesting is challenging and inefficient, as only 0.001–0.01% of bone marrow cells are MSCs (27, 28). The risk of infection during the isolation of cells from the bone marrow also cannot be ignored. Consequently, a more effective and less invasive procedure is required, and scientists have attempted to identify new extraction sites (27, 28).

In 2000, human umbilical cord blood was recognized as an alternative source of MSCs (29). Umbilical cord blood-derived MSCs (UCB-MSCs) show rapid self-renewal and differentiation capabilities, thereby promoting tissue repair and modulation of immune responses; moreover, these cells are easy to access with painless extraction procedures (30). UCB-MSCs have rapid proliferation rates that are approximately three- to four-fold greater than that of adipose tissue (AT)-MSCs (31, 32). Furthermore, UCB-MSCs are known to secrete multiple growth factors associated with skin rejuvenation, such as epithelial growth factor (EGF), collagen type 1, hepatocyte growth factor (HGF), and growth differentiation factor-11 (GDF-11) (33). Indeed, UCB-MSCs have been reported to possess anti-wrinkling effects and the ability to increase dermal density. Because of these benefits, researchers claim that the clinical applications of UCB-MSCs extend beyond the limits of those of BM-MSCs (34). However, previous studies have also reported undesirable characteristics of UCB-MSCs, such as earlier morphological changes and faster loss of amplification ability, along with lower attachment efficiency (31, 35, 36).

Human AT-MSCs were identified as another promising source of MSCs in 2001, because of its accessibility and abundancy as well as its stronger immunosuppressive effects. Unlike BM-MSCs, AT-MSCs can be extracted in large, concentrated quantities (about 500 times more than BM-MSCs) using relatively simple procedures and local anesthesia (37). Another benefit of AT-MSCs is that they can be extracted from various human body sites; however, AT-MSCs extracted from different sites have shown varied traits (38). For instance, Nepali et al. concluded that orbital AT-MSCs have higher expressions of CD73, CD90, CD105, and CD146, but lower expressions of CD31, CD45, and HLA-DR, than abdominal AT-MSCs (38). Moreover, Kim et al. reported increased expression of HLA-ABC and HLA-DR in AT-MSCs after IFN-γ treatment, raising concerns about the application of allogenic AT-MSCs (32). Hence, more research investigating donor-matched AT-MSCs from different isolation sites and their respective traits is required to fully understand the defining phenotypes and increase the clinical efficiency of these MSCs (28, 38).

While the previously mentioned sites represent the most common tissues for MSC extraction, the synovial membrane also contains MSCs. Synovial membrane-derived MSCs (SM-MSCs) were first isolated in 2001 by De Bari et al. (39). Like AT-MSCs, SM-MSCs can be extracted from various sites, including the cotyloid fossa or paralabral synovium, with site-specific traits (40). Interestingly, SM-MSCs have extensive proliferative ability, multilineage differentiation potential, and low immunogenicity relative to other MSCs (39, 41). Due to higher expression of type II collagen, aggrecan, and SRY-box transcription factor 9 in SM-MSCs, they have demonstrated higher chondrogenic potential than MSCs from other sources and are expected to be more widely used for cartilage repair and joint homeostasis treatments (42–44). Moreover, a study by Sakaguchi et al. concluded that SM-MSCs and BM-MSCs have greater osteogenic and adipogenic potentials than other MSCs; however, SM-MSCs foster relatively low-density expansions *in vitro* compared to BM-MSCs (41, 45).

## Applications of MSC Therapies for Cartilage Injuries

The safety and efficacy of MSCs in the treatment of joint-related diseases and cartilage injuries have been continuously examined over the recent decades. Concurrently, the prevalence of cartilage lesions have also significantly increased during this period, as the early incidence rates of this condition have roughly tripled from 1996 to 2011 (46, 47). Despite the high prevalence rate, a universally efficient method for articular cartilage repair is yet to be developed (48). Current surgical options include arthroplasty, microfracture, and autologous chondrocyte implantation (49). The promising qualities of MSC-based therapies could potentially provide effective, less invasive procedures to repair articular cartilage defects. In an experimental trial, BM-MSCs were transplanted into the patellae of two patients with full-thickness articular cartilage defects (50). Two years after transplantation, the arthroscopic results showed significant improvements in the walking abilities of both patients (50). Similarly, another case study involving a judo athlete diagnosed with a full-thickness cartilage defect in the medial femoral condyle exhibited recovery within months after the implantation of MSC-embedded collagen gel with reduced pain (51). Furthermore, a 2010 study compared the clinical outcomes of cartilage lesion repair between implantations of first-generation autologous chondrocytes and BM-MSCs in groups of 36 patients each; all patients showed improvements in quality of life with no significant differences between the groups (52). Therefore, it was concluded that BM-MSC treatment is a cost-efficient option for cartilage lesion repair with minimal donor-site morbidity and fewer surgical procedures than autologous chondrocyte implantation (52). Thus, multiple clinical trials have revealed the promising potential of MSC therapy in cartilage repair.

**Figure 2:**
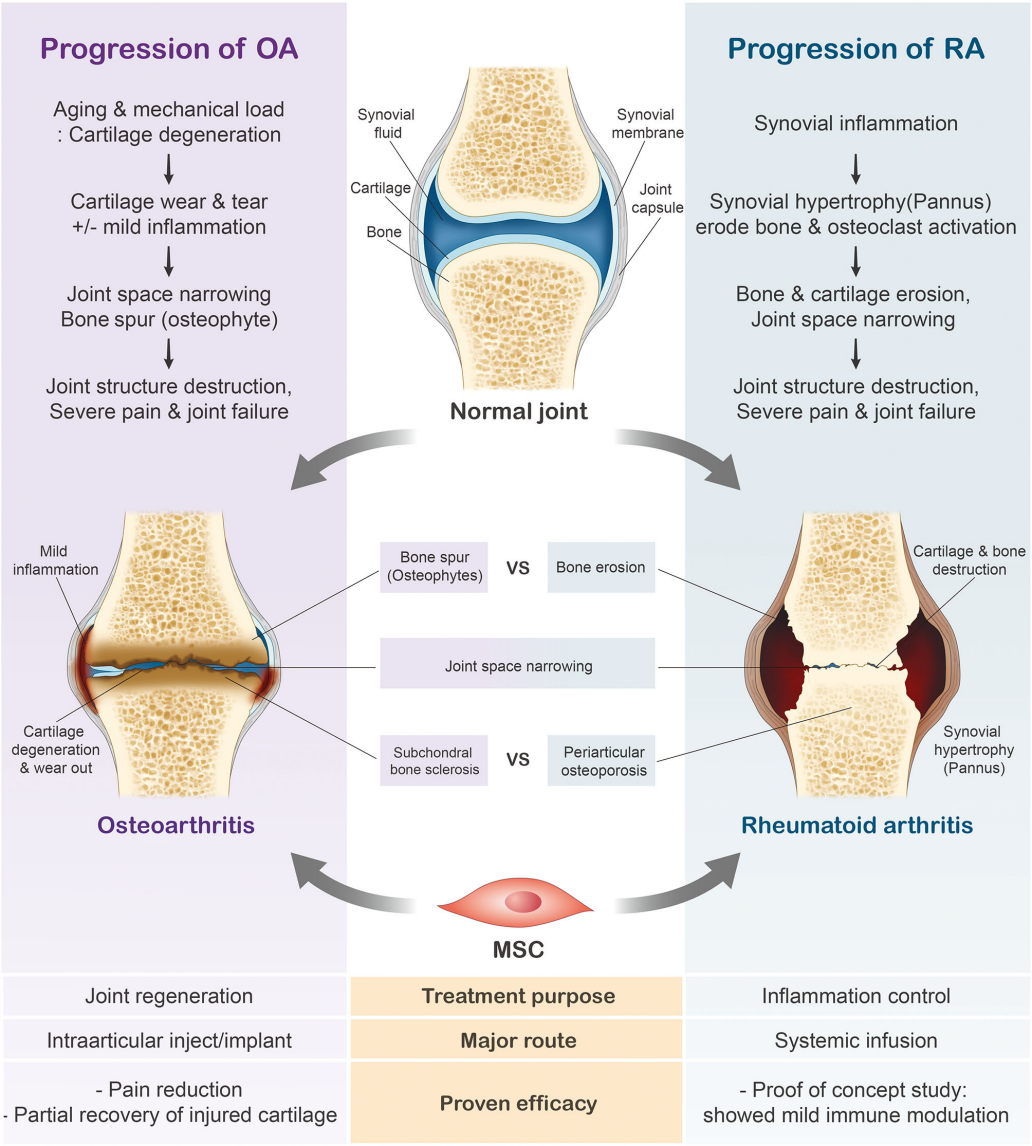
Progression of rheumatoid arthritis (RA) and osteoarthritis (OA). In contrast to the healthy joint, the osteoarthritic joint has thinned cartilage and the bone ends rub together. Joints diagnosed with RA have swollen, inflamed synovial membranes and undergo bone erosion. The cartilage erosion seen in the later stages of arthritis ultimately leads to the loss of mobility.

Variations in the general characteristics of rheumatoid arthritis (RA) and osteoarthritis (OA) largely depend on their etiologies and initial symptoms. RA is a chronic systemic autoimmune disease characterized by joint inflammation and bone erosion (53, 54), whereas OA is a degenerative joint disease that triggers the gradual loss of articular cartilage (55, 56). While increased bone spur growth is observed in osteoarthritic joints during the early stages, RA joints initially experience synovial inflammation (Figure 2). Ultimately, patients diagnosed with either joint condition suffer from severe cartilage inflammation and loss of mobility (56, 57). In this review, we will further discuss the use of MSCs in these two respective joint diseases.

## Applications of MSC Therapies in OA

### General Characteristics of OA

OA is the most common degenerative joint disease (55). Its initial symptoms include loss of articular cartilage followed by progressive to joint stiffness, swelling, pain, and loss of mobility (56). The prevalence rate of OA is extremely high, affecting more than 250 million people worldwide (58–60). With increases in the aging and obese populations, the prevalence of OA is predicted to increase to 67 million by 2030 (61). Hence, an effective and safe OA treatment is urgently required.

Like RA, OA is also divided into two groups based on its etiology: primary and secondary (59). Primary OA is idiopathic and gene-dependent, whereas secondary OA mainly originates from traumatic events (58, 62, 63). Despite different etiologies, the two types of OA progress in similar directions, ultimately resulting in the loss and destruction of articular cartilage (55).

Although aging is one of the biggest risk factors for OA, the underlying mechanisms and related factors are yet to be definitively established (55, 56, 64). An imbalance in the production and activities of catabolic mediators in aging cells is a cause of the destruction and loss of articular cartilage (65). A disturbed ratio of the transforming growth factor β (TGF-β) receptors activin receptor-like kinase 1 (ALK1) and ALK5 triggers the downregulation of the TGF-β pathway and induces matrix metalloproteinase (MMP) expression, which degrades structural proteins in the cartilage (55, 66). The exact reason for this imbalance in signaling is assumed to be the senescent phenotype of OA chondrocytes, but a clear explanation is still required (65). Age-related mitochondrial dysfunction has also been suggested to promote the development of OA (66, 67). Aged articular chondrocytes and other cells display increased secretion of reactive oxygen species (ROS), and thereby elevated oxidative stress (67–69). The production of ROS ultimately alters mitochondrial function, leaving them unable to synthesize proteoglycans, the primary building blocks of the cartilage extracellular matrix (ECM) (70).

Genetic predisposition is another major risk factor of primary OA, with almost 30–65% of OA risk being genetically determined (56, 64, 71, 72). Recent genome-wide association studies (GWAS) have drastically expanded our understanding of the genetic risk factors of OA (71, 73). Currently, 90 loci are known to pose significant OA genetic risks, and 80 possible gene mutations or single nucleotide polymorphisms (SNPs) have been identified to be involved in OA pathogenesis (74). These include genes encoding other structural factors (*Col2a1, Col9a1*, and *Col11a1*) and bone morphogenetic proteins (*Gdf5*) (75–81). There are various ongoing studies focused on fully uncovering the genetic risk factors of OA. Most notably, a 2019 study analyzed ~77,000 patients with OA and 378,000 undiagnosed individuals from the UK Biobank cohort to identify 52 novel OA-associated signals (74). While most studies have specifically investigated OA susceptibility loci in Europeans or those of European descent, there have also been studies targeting other populations (82–85). A 2020 study by Zhao et al. showed a significant correlation between the SNP rs10896015 in the *LTBP3* gene and hip OA among the Chinese population (86). Furthermore, another study revealed that the SNP rs4238326 in the *ALDH1A2* gene, which was previously reported to trigger hand OA in European populations, is also linked to knee OA risk (86, 87).

Obesity has become a highly prevalent disease in contemporary society and it is estimated to spread to almost 20% of the global population by 2030 (88). Obese patients with unhealthy diets are exposed to multiple risk factors of OA, as one study reports a 24% increase in the likelihood of developing OA in obese individuals compared to those of healthy weight (89). First and foremost, mechanical overload in joints promotes ROS production by OA chondrocytes, which further aggravates cartilage degradation (90). A recent study concluded that there is a 2.5-times higher likelihood of patients with diabetic knee OA experiencing knee pain compared to patients with non-diabetic knee OA due to accelerated cartilage damage (91). Furthermore, obesity has also been associated with the secretion of adipokines, thus contributing to low-grade systemic inflammation (92–95). The expression levels of pro-inflammatory cytokines, such as interleukin (IL)-1β, IL-6, IL-8, and tumor necrosis factor-α (TNF-α), are increased in obese individuals (96–101). Inflammatory factors activate the nuclear factor-κB (NF-κB) signaling pathway and ultimately result in the upregulation of MMPs, subsequently targeting the ECM (102–104). Lastly, meta-analysis studies have shown a significant relationship between obesity and dose-response. Patients with higher body mass indices are less likely to be dose-responsive and show continual clinical consequences (89, 105, 106).

Although moderate physical activity is encouraged to improve one's general health, repetition or incorrect execution of movements is the prevailing cause of OA in both young and older adults (56, 64, 107). From a comprehensive review of recent studies, common occupational activities such as sitting (hip and knee OA), lifting (knee OA), driving (knee OA), and squat (lower limb OA) have been associated with increased risk of OA (108, 109). These activities, if repeated, could be detrimental to the joint, as a study calculated the risk of developing localized OA to be twice as likely in individuals with occupations demanding repetitive physical activities compared to the average population (110). Moreover, although recreational sports activities are known to lower the occurrence of OA, elite athletes participating in competitive sports are extremely prone to OA, as incorrect execution of movements may disturb joint stabilization (111–114). In a systematic review including over 8,400 subjects, it was concluded that soccer, weightlifting, and wrestling were the sports with the highest prevalence of knee OA (112). Furthermore, it has been reported that OA prevalence rates in contact sports, such as rugby, are twice as high as those in non-contact sports (113).

### Clinical Trials Using MSCs in OA

We also reviewed recent clinical trials that used MSC-based treatments in patients with OA (Table 2). A clinical trial by Kim et al. involved 49 patients (55 knees) with isolated full-thickness cartilage lesions and Kellgren-Lawrence (K-L) grade 1 or 2 OA with symptoms of knee joint pain and/or functional limitations despite non-surgical treatments for a minimum of 3 months (115). AT-MSCs were harvested from each patient's buttocks via tumescent liposuction. Upon isolation, AT-MSCs were loaded into a fibrin glue product and surgically implanted into the lesion site. Implanted knees were immobilized with a knee brace for 2 weeks post-surgery, followed by passive joint exercises. During the follow-ups, it was reported that the mean International Knee Documentation Committee (IKDC) score increased from 37.7 to 67.3, and the Tegner Activity Scale from 2.2 to 3.8. Both scores showed significant improvements in patients, with 74.5% of them expressing better satisfaction. In addition, age and lesion size were identified as independent factors affecting clinical outcomes. Patients over 60 years of age with lesion sizes >6.0 cm^2^ showed less favorable results. Although there were some variations in the results due to these factors, the overall clinical outcomes of MSC implantation in OA patients were encouraging, with successful results.

**Table 2:** Clinical trials using MSCs in OA.

**Year**	**References**	**Sample**	**Source of MSC**	**Injection method**	**Treatment group**	**Result**
2015	Kim et al. (115)	49 patients	AT-MSCs	Loaded into fibrin glue product to be surgically implanted into lesion site	Patients received approximately same amount of MSC (4.3 × 10^6^) via arthroscopic procedure	Patients showed overall satisfaction with improved mean IKDC and Tegner activity scores. Regarding the efficacy of MSC implantation, it was concluded that there was a cutoff for both age (>60 years old) and cartilage lesion size (>6.0 cm^2^)
2016	Shapiro et al. (116)	25 patients	BM-MSCs	Combined with platelet-poor plasma for injection	25 patients were randomly divided into two groups. 12 patients had BMAC injected in their left knee and placebo in their right, while 13 patients were injected BMAC on their right knee and placebo in their left. The BMAC product had a median of 34,400 MSCs	Significant improvements in ICOAP scores, VAS pain scores, activity level, and pain medication usage were observed from both placebo and treated knees. No adverse events were reported which ensured the safety of MSC treatment
2016	Pers et al. (117)	18 patients	AT-MSCs	Intra-articular injection in the knee joint	18 patients were divided into 3 cohorts with increasing dosage: 2 × 10^6^ (low dose), 10 × 10^6^ (medium dose), and 50 × 10^6^ (high dose)	Only the low dosage group showed statistically significant improvements in all categories of WOMAC index, VAS pain score, and KOOS index. The medium dose group showed improvements in some categories. The high dose group did not have any statistically significant results. Thus, there was an inverse dose effect
2018	Matas et al. (118)	26 patients (with 8 serving as controls)	UCB-MSCs	Intra-articular injection	Patients in the control group received hyaluronic acid treatment and MSC-treated patients were divided into two groups (*n* = 9). The first group received single dose of UCB-MSC (20 × 10^6^), while the second group received two dosages (20 × 10^6^) 6 months apart	Some patients in MSC treated groups showed acute synovitis after injection. No serious adverse events were reported. Improvements in pain and function with lower WOMAC and VAS pain scores was observed compared to the control group without any differences in MRI scores
2019	Freitag et al. (119)	30 patients (with 10 serving as controls)	AT-MSCs	Intra-articular injection	Patients were separated equally into three groups (*n* = 10). The control group continued to receive conventional conservative management. The first treatment group received one MSC injection (1 × 10^8^ AT-MSCs). The second treatment groups received two injections 6 months apart (1 × 10^8^ AT-MSCs)	The two treated groups saw significant reduction in pain measured by NPRS and WOMAC scores. MSC injection was also concluded to reduce the rate of cartilage loss upon MRI analysis. Although minor discomfort and bruising was common for treated groups, no serious adverse events were reported
2019	Chahal et al. (120)	12 patients	BM-MSCs	Intra-articular injection	Patients were divided into four cohorts (*n* = 3). Each group received a single intra-articular injection of BM-MSCs. The first three cohorts received (1 × 10^6^, 10 × 10^6^, and 50 × 10^6^ of BM-MSCs) The fourth cohort had each patient receive the different dosages of MSC listed above	Although four patients reported pain and swelling after injection, no other serious adverse events were reported. Patients who received higher dosages of MSCs saw more significant improvements in KOOS, WOMAC stiffness, quality of life, and symptoms
2019	Lee et al. (121)	24 patients (with 12 serving as control)	AT-MSCs	Intra-articular injection	Patients in the treated group received inter-articular injection of AT-MSC (1 × 10^8^) in 3 mL of saline	The MSC treated group showed significant improvements in WOMAC and VAS scores. Furthermore, the size of the cartilage defect was increased in the control group, while no significant change was observed in the MSC group. No serious adverse events were reported

In 2016, Shapiro et al. conducted a randomized, single-blinded, placebo-controlled trial in 25 patients with mild to moderate bilateral knee OA who had previously received conventional treatments, such as activity modification or physical therapy (116). Each patient had ~52 mL of bone marrow harvested from their respective superior iliac crests. The marrow cells were then analyzed for the positive and negative co-expression of surface markers to fulfill the minimal criteria for defining MSCs. Upon confirmation, 5 mL of cells were mixed with 10 mL of previously separated platelet-poor bone marrow plasma to be injected into a randomly assigned knee of each patient (13 patients received MSCs in their right knee, and 12 patients in their left knee). The contralateral knees subsequently underwent an intra-articular injection of 15 mL of sterile saline, and served as controls. After 1 week, 3 months, and 6 months, the Measure of Intermittent and Constant Osteoarthritis Pain (ICOAP) questionnaire and visual analog scale (VAS) pain scores of the two groups were recorded. The VAS pain scores and answers to the ICOAP questionnaires indicated significant improvements throughout the follow-up periods in both the bone marrow aspirate concentration group and the placebo group. Furthermore, both groups showed increased activity levels and decreases in self-reported pain medication usage, with no difference in the degree of improvement from baseline. The efficacy of MSC injection was questioned, as there was no difference in pain-mediating capabilities between the knees treated with the BM-MSC injection vs. saline.

In 2016, Pers et al. published a phase I, prospective, bicentric, single-arm, open-label, dose-escalating clinical trial report of AT-MSC injection in patients with knee OA (117). The 18 patients selected for the trial were 50–75 years of age with K-L grade 3–4 knee OA. The subjects were first divided into three consecutive cohorts with increasing dosages: 2 × 10^6^ (low dose), 10 × 10^6^ (medium dose), and 50 × 10^6^ (high dose) cells. The primary outcome assessed the safety of the trial, while the secondary outcome measured clinical efficacy. The AT-MSCs were extracted from the respective patients through liposuction and the prepared AT-MSC dosages were administered via intra-articular injections to the knee joints. In the primary outcome assessment, no adverse events from either liposuction or intra-articular injection were observed. However, one patient who had hypertension and hyperlipidemia suffered from unstable angina pectoris without increased levels of cardiac markers 3 months after treatment. In addition, four other patients reported minor knee pain/joint effusion that resolved spontaneously or after treatment with non-steroidal anti-inflammatory drugs. Thus, the safety of AT-MSC treatment was further demonstrated. The secondary outcome was initially assessed using magnetic resonance imaging (MRI), which showed no correlation between MRI and clinical results, in addition to histologic analysis that showed no indication of tumor proliferation. In contrast to other studies, only the low dosage group presented statistically significant results in all categories of the Western Ontario and McMaster Universities Osteoarthritis (WOMAC) index, VAS pain score, and Knee Injury and Osteoarthritis Outcome Score (KOOS). This inverse dose effect could possibly be due to increased inflammation in the low-dose group. Despite limited resources and unclear explanations, Pers et al. further demonstrated the safety and promising potential of MSC treatment.

Matas et al. led a randomized, double-blinded, controlled clinical trial including 29 patients aged 40–65 years with K-L grade 1–3 knee OA (118). Patients were divided into three groups and received two injections 6-months apart. The hyaluronic acid (HA) group (control) received two HA injections (3 mL of Durolane). The MSC-1 group received UCB-MSC (2 × 10^7^ UCB-MSCs and 5% AB plasma in 3 mL of saline) at baseline and was later injection with placebo (5% of AB plasma in 3 mL of saline), while the MSC-2 group received two UCB-MSC injections (2 × 10^7^ UCB-MSCs and 5% AB plasma in 3 mL of saline). Although acute synovitis was common after UCB-MSC injections, no serious adverse events were observed during the trial. Clinical assessment revealed that the MSC-2 group had significantly lower total WOMAC indices than the HA control group at 12 months (4.2 vs. 15.2). In parallel with this result, the VAS score of the MSC-2 group was 2.4, while the VAS score of the HA group was 22.1. In contrast to the MSC-2 group, the MSC-1 group did show improvements through the first 9 months, but later became ineffective after receiving an HA injection. Overall, no patients in any group showed evidence of chondral damage or intra-articular calcifications upon MRI follow-up. The clinically significant results indicated that repeated administration of UC-MSCs dosage is a favorable and safe means of improving the clinical outcomes of patients with knee OA.

Freitag et al. conducted a randomized trial involving 30 patients aged 18 years or older with K-L grade 2–3 knee OA who had previously undergone primary conservative management of OA, such as weight management programs and bracing (119). The participants were first randomly divided into three groups: two treatment groups and one control group. The first group was injected once with 1 × 10^8^ MSCs, while the second group received two injections of 1 × 10^8^ MSCs 6 months apart. The third group served as the control with continued conservative treatments. MSCs were harvested from the adipose tissues of the patients and cultured until passage 2 prior to injection. AT-MSCs were then injected under ultrasound guidance into the intra-articular knee space. The primary outcomes measured the pain and functional changes after the procedure; the secondary outcome involved an MRI analysis after 12 months. Relative to the control, the two treated groups showed significant improvements in pain according to the numeric pain rating scale (NPRS) (6.7 and 6.5 to 2.6 and 2.3) and WOMAC scores (59.6 and 54.4 to 84 and 87.3). MRI analysis showed that 37% of the participants in the first treatment group exhibited further cartilage loss compared to the control. However, ~89% of the patients in the second treatment group showed marked improvement or no progression in cartilage loss. Furthermore, no serious adverse events were observed in the two treated groups during follow-up. Thus, it was concluded that AT-MSC therapy is a safe and effective treatment for knee OA and that frequent injections are preferable.

In 2019, Chahal et al. published a non-randomized, open-label, dose-escalation clinical study (120). This study included patients aged 40–65 years, who were diagnosed with K-L grade 3–4 knee OA and had failed to derive benefits from non-operative treatment regimens for at least 6 months. A total of 12 patients were divided into four cohorts. The first three cohorts were injected with 1 × 10^6^, 2 × 10^7^, and 5 × 10^7^ BM-MSCs (extracted from the posterior superior spine, respectively). In the fourth cohort, three patients each received different BM-MSCs dosages consistent with the increasing dosage levels injected into the three previous cohorts. The primary outcome ensured the safety of the trial, and the secondary outcomes involved clinical, radiological, and biomarker assessments. Without any adverse events, patients saw significant improvements in KOOS and WOMAC stiffness scores, quality of life, and symptoms 12 months post-BM-MSC treatment. Moreover, patients treated with higher dosages demonstrated better chances of significant improvements compared to those treated with lower dosages. Hence, it was concluded from this study that BM-MSC treatment is safe with positive clinical outcomes, specifically at higher dosages.

In 2019, Lee et al. conducted a randomized, double-blinded, placebo-controlled study in 24 patients with knee OA aged 18–75 years, who had a mean pain intensity (VAS score) of 4 or higher for a minimum of 12 weeks with at least one grade 3–4 lesion (121). Patients were randomly divided into MSC and control groups. MSCs were then isolated from the abdominal subcutaneous adipose tissue via lipoaspiration. After the isolated AT-MSCs were cultured to passage 3, the MSC group was treated with an intra-articular injection of 1 × 10^6^ AT-MSCs in 3 mL of saline, while the control group was injected with 3 mL of saline alone. The primary clinical outcome was evaluated 6 months after injection using the WOMAC index. In addition, the secondary outcomes comprised clinical scores, physical examination, radiological evaluation, and safety assessment. In the follow-up, the control group showed no drastic changes in any of the outcomes. On the contrary, the MSC group showed significant reductions in WOMAC and VAS scores for knee pain, from 60.0 to 26.7 and 6.8 to 3.4, respectively. Moreover, the physical and radiological examination data showed that the MSC-injected patients demonstrated a wider range of knee motion (127.9°-134.6°) and unchanged cartilage defects, in contrast to the enlargement seen in the control group. Finally, with all adverse events below grade 3 of the National Cancer Institute-Common Terminology Criteria for Adverse Events scale, the use of intermittent acetaminophen could remediate all treatment-related adverse events without any treatment discontinuations. Although some evaluations (K-L grade, HKA angle, quadriceps power, and the presence of joint effusion) did not show any difference or improvement between the MSC and control groups, it was concluded that the intra-articular injection of AT-MSCs resulted in satisfactory clinical and functional outcomes without serious short-term safety concerns.

## Applications of MSC Therapy in RA

### General Characteristics of RA

RA is a chronic systemic autoimmune disease that causes progressive disability and premature death (53). This disease initially affects the synovial joints and later progresses to the skin, eyes, heart, kidneys, and lungs (53, 57). Ultimately, the patient suffers from joint failure characterized by cartilage damage and severely weakened tendons and ligaments (57, 122). The prevalence rate of RA was reported to be ~0.5–1.0% across the global population in 2002, with females being twice as more likely to be affected due to unknown factors (123, 124).

Based on the presence or absence of anti-citrullinated protein antibodies, RA is divided into two major subtypes (53), which show significant differences in their respective genomes and have completely different pathophysiologies (125). The primary genetic risk factors of RA include alleles encoding the HLA-DR region (53, 126–130). Other critical components are environmental risk factors, such as exposure to tobacco smoke, and lifestyle factors, such as dietary habits (53, 128). As the mechanisms of RA development and its specific targeting of the joints remain unclear, further research is required to fully understand this process (53). The fulminant stage of RA involves hyperplastic synovium, cartilage damage, and bone erosion (53). Along with bone loss, both inflammation and autoimmune responses are potential causes of RA progression (53). This cascade of reactions is activated when fibroblast-like synoviocytes (FLSs) interact with immune cells of the innate and adaptive immune systems (53). Some of the immune cells responsible for inflammation are monocytes, macrophages, T lymphocytes, and B cells (53, 131, 132). The synovial membrane and cartilage undergo significant inflammation, causing hyperplastic synovium and cartilage destruction that eventually lead to bone erosion (54). Hyperplastic synovium is a critical characteristic of RA, and there are two hypotheses regarding its cause. The first is that the abnormal proliferation of FLSs ultimately leads to the production of inflammatory cytokines and mediators that continue joint destruction (53, 133). The second is that the resistance to apoptosis due to defects in tumor protein p53 triggers the hyperplastic synovium (53, 134). Here, the shortage of chondrocytes caused by apoptosis would result in cartilage degeneration and joint-space narrowing via directed adhesion and invasion (53, 135, 136).

### Clinical Trials Using MSCs in RA

Compared to OA, relatively few trials have been performed in RA with MSCs. In some cases, it is said that MSC therapy is not suitable for RA given the growing armamentarium of other efficient therapeutic agents available in contrast to OA. Systemic administration of autologous MSCs seemed to cause an exacerbation of RA in a collagen-induced arthritis (CIA) RA animal model, whereas the results of administration of allogeneic MSCs were more successful. These results suggest that allogeneic MSCs are more effective in treatment for autoimmune disorders (137, 138). Although there have been a limited number of clinical trials of MSC treatment in RA patients, the safety and efficacy of therapy have been confirmed in several studies (139). Here, we have briefly reviewed recent clinical trials that used MSC-based treatments in patients with RA (Table 3).

**Table 3:** Clinical trials using MSCs in RA.

**Year**	**References**	**Sample**	**Source of MSC**	**Injection method**	**Treatment group**	**Result**
2013	Wang et al. (140)	172 patients (with 36 patients serving as control)	UCB-MSCs	Intravenous injection	136 patients were divided into three groups based on the interval after the first injection Group 1 (*n* = 76): 3 month-interval Group 2 (*n* = 45): 6 month-interval Group 3 (*n* = 15): 8-month interval (4 × 10^4^ cells per injection)	MSC injections with DMARDs treatment lowered the HAQ and DAS28 scores in 3–6 months follow-up compared to the control group who had only received DMARDs
2018	Park et al. (141)	9 patients	UCB-MSCs	Single intravenous infusion	Nine patients were divided into three groups depending on their injection dosage: 2.5 × 10^7^, 5 × 10^7^, or 1 × 10^8^	No adverse events were recorded. Lower VAS and DAS28 scores were reported in patients who received higher dosages
2020	Ghoryani et al. (142)	13 patients	BM-MSCs	Single intravenous injection	13 patients each received a single intravenous injection of autologous BM-MSCs (1 × 10^6^ per kg)	During the 12-month follow-up period, increased FOXP3, IL-10, and TGF-β1 expression were observed leading to a conclusion that BM-MSC treatment has immunoregulatory effects on regulatory T cells of RA patients

Wang et al. conducted a randomized controlled clinical trial with 172 patients with RA who previously underwent unsuccessful chemotherapy treatments and were currently prescribed disease-modifying anti-rheumatic drugs (DMARDs) (140). The MSC-treated group (*n* = 136) received 4.0 × 10^7^ UC-MSCs in 40 mL of stem cell solvent, while the control group (*n* = 36) received only 40 mL of stem cell solvent via intravenous infusion. The MSC-treated group was then further divided into three groups based on the intervals after the first treatment: Group 1 had a 3-month interval, Group 2 had a 6-month interval, and Group 3 had an 8-month interval between injections. The safety of the trial was assessed through radiographic and physical examinations, while disease activity was monitored via disease activity score 28 (DAS28) and the Health Assessment Questionnaire (HAQ). This study did not find serious side effects other than minor fevers and chills. Two weeks after the intravenous injection, the MSC-treated groups showed higher quality of life with reduced joint pain/swelling compared to that of the control group. Moreover, decreased DAS28 and HAQ scores were recorded in the MSC-treated group with repeated treatments, indicating a steady reduction in disease activity. Thus, treatment with a combination of DMARDs and UCB-MSC via injection was concluded to be safe and effective in reducing the long-term disease activity of refractory RA compared to that of conventional DMARDs treatment alone.

Park et al. conducted a clinical trial to test the safety of short-term application of UCB-MSCs in patients with RA (141). The nine participating patients, all aged 18 years or older, had baseline DAS28 assessments and were on a stable dose of methotrexate for a minimum of 12 weeks. Patients received different concentrations of UCB-MSCs via intravenous infusion. Follow-ups for the assessment of clinical and safety parameters were conducted 24 h, 3 days, 1 week, and 4 weeks after infusion. At 4 weeks post-infusion, no abnormalities were detected in the hematologic and chemical profiles; only minor elevations in serum uric acid levels were observed among patients. Hence, it was reported that there were no serious adverse events or dose-limiting toxicities due to the application of UCB-MSCs. UCB-MSC treatment reduced the disease activity of RA and dose-dependently reduced the DAS28 score and VAS pain scale.

Ghoryani et al. conducted a clinical trial to test the immunoregulatory effects of MSCs on 13 female patients with refractory RA, who had previously received maximum dosages of DMARDs (142). The patients had their respective BM-MSCs transplanted via a single dose (1 × 10^6^ per kg of body weight) and were followed up at 1, 6, and 12 months post-transplantation. Patients showed a significant reduction in DAS28 score (from 5.56 to 4.72) after 12 months of MSC treatment. Increased forkhead box P3 (FOXP3), IL-10, and TGF-β1 gene expressions were observed in patients treated with MSCs. Based on the increases in IL-10 and TGF-β, MSC therapy was concluded to have significant immunomodulatory effects in patients with refractory RA. Nevertheless, further research is required to investigate the possible effects of increasing/replicating the MSC dosages in patients for improved results.

## Strategies for Future Use of MSCs

The terminology debate over “stem” versus “stromal” has been argued in the past and is still ongoing (9). In 2005, the ISCT committee issued a paper that clarifies that the term MSC is not equivalent (or interchangeable) with mesenchymal stromal cell (143). While the MSCs previously discussed in our study refers to cells with self-renewal and differentiation, mesenchymal stromal cells refers to a bulk population of cells with secretory, immunomodulatory effects with additional homing ability (144–146). This is a critical point in studies using MSCs, as the ISCT MSC committee recommends to clarify whether MSC stands for “mesenchymal stromal cells” or “mesenchymal stem cells.” However, currently, there is no surface marker that can be used to distinguish these two cell types. The ISCT MSC committee endorses the functional distinction between stromal and stem cells and suggests further analysis focused on their functionalities along with their secretomes. With advanced analysis at the single cells levels and mass cytometry using next-generation sequencing tools, it is important to distinguish the epigenomic, transcriptomic, and proteomic differences between the mesenchymal stromal cells and stem cells. Future studies that target treatment and regeneration of the defected joint tissue should consider thoroughly characterizing the attributes along with the stemness of the MSCs that are used in each study. Such detailed characterization of the investigated MSCs may suggest a unique subtype of MSCs for more direct targeting for the treatment of arthritic diseases.

## Conclusion

In this review, we have summarized the current status of MSC therapies for OA and RA. While, OA had more promising studies and results compared to that of RA, MSC therapy has shown potential in both OA and RA treatments with reduced pain, improved joint function, and enhanced overall life satisfaction in patients. Clinical trials on OA and RA discussed in this review demonstrate that MSCs are a safe treatment option without serious adverse events. However, more studies are required to examine the long-term safety of MSC injections and their respective clinical applications. Future research studies employing the latest in technology can be the key to increasing scientific evidence concerning their efficacy and safety of MSC therapies. In addition, thorough examination and characterization of the studies already using MSCs are critical for the better understanding of MSCs and will allow them to become a leading candidate for the treatment of various diseases, including arthritic diseases.

## Author Contributions

JH and JJ concepted the topic, collected data, and wrote the manuscript. YR and YN reviewed and revised the work. JJ supervised this project. All authors contributed to the article and approved the submitted version.

## Conflict of Interest

The authors declare that the research was conducted in the absence of any commercial or financial relationships that could be construed as a potential conflict of interest.

## Abbreviations

ACPA: anti-citrullinated protein antibodies
AI: arthritis index
AIA: antigen-induced arthritis
ALK: activin receptor-like kinase
AT-MSC: adipose tissue-derived mesenchymal stem cell
BMAC: bone marrow aspirate concentration
BM-MSC: bone marrow-derived mesenchymal stem cell
CD: cluster of differentiation
DAS28: disease activity score
DMARD: disease-modifying anti-rheumatic drug
ECM: extracellular matrix
FLS: fibroblast-like synoviocytes
GFP-MSC: green fluorescent protein-positive mesenchymal stem cells
HA: hyaluronic acid
HAQ: health assessment questionnaire
HKA angle: hip-knee-ankle angle
HLA: human leukocyte antigen
ICOAP: pain questionnaire, measure of Intermittent and Constant Osteoarthritis Pain questionnaire
ICST: International Society of Cellular Therapy
IFN- γ: interferon gamma
IGF-1: insulin-like growth factor 1
IKDC: International Knee Documentation Committee
IL: interleukin
K-L grade: Kellgren-Lawrence grade
KOOS: Knee Injury and Osteoarthritis Outcome Score
MMP: matrix metalloproteinases
MRI: magnetic resonance imaging
MSC: mesenchymal stem cell
NPRS: Numeric Pain Rating Scale
NF-κB: nuclear factor-kappa B
OA: osteoarthritis
OP-1: osteogenic protein 1
PGA: patient global assessment
RA: rheumatoid arthritis
ROS: reactive oxygen species
GWAS: genome-wide association studies
SF-36: Questionnaire, 36-Item Short Form Survey
SM-MSC: synovium-derived mesenchymal stem cell
SNP: single nucleotide polymorphism
TNF: tumor necrosis factor
UCB-MSC: umbilical cord blood-derived mesenchymal stem cell
VAS: visual analog scale
WOMAC index: Western Ontario and McMaster Universities Osteoarthritis index
WORMS: whole-organ MRI scoring.
